# Longitudinal Transitions of Metabolic-Obesity Phenotypes and Subsequent Cardiovascular Disease Risk: A Prospective Analysis of the CHARLS Cohort

**DOI:** 10.3390/metabo16070510

**Published:** 2026-07-21

**Authors:** Wenjing Yan, Xiaona Zhang, Qingqing Man, Shanshan Jia, Wenjing Feng, Lili Chen, Rongzhen Li, Lianlong Yu, Liangkai Chen, Jian Zhang, Pengkun Song

**Affiliations:** 1 Department of Elderly Nutrition and Clinical Nutrition, National Institute for Nutrition and Health, Chinese Center for Disease Control and Prevention, Beijing 100050, China; 15890793674@163.com (W.Y.); zhangxn@ninh.chinacdc.cn (X.Z.); manqq@ninh.chinacdc.cn (Q.M.); jiass@ninh.chinacdc.cn (S.J.); fengwj@ninh.chinacdc.cn (W.F.); chenll@ninh.chinacdc.cn (L.C.); zhangjian@ninh.chinacdc.cn (J.Z.); 2 NHC Key Laboratory of Public Health and Nutrition, Beijing 100050, China; 3 NHC Specialty Laboratory of Food Safety Risk Assessment and Standard Development, Guangdong Provincial Center for Disease Control and Prevention, Guangzhou 511430, China; yc07606@um.edu.mo; 4 Shandong Center for Disease Control and Prevention, Shandong Provincial Academy of Preventive Medicine, Jinan 250014, China; lianlong00a@163.com; 5 Department of Nutrition and Food Hygiene, School of Public Health, Huazhong University of Science and Technology, Wuhan 430030, China; clk@hust.edu.cn

**Keywords:** metabolic-obesity phenotypes, longitudinal transitions, cardiovascular disease, CHARLS, prospective cohort study

## Abstract

**Background/Objectives**: Metabolic-obesity phenotypes are dynamic, yet little is known about how their longitudinal transitions affect cardiovascular disease (CVD) risk. We aimed to characterize these transitions and examine their associations with incident CVD in middle-aged and older Chinese adults. **Methods**: We included 4516 participants from the China Health and Retirement Longitudinal Study (CHARLS) who were free of CVD at the follow-up baseline (wave 3). Metabolic-obesity phenotypes (metabolically healthy non-obese [MHNO], metabolically unhealthy non-obese [MUNO], metabolically healthy obese [MHO], and metabolically unhealthy obese [MUO]) were assessed in 2011 and 2015, and six trajectory patterns were defined. Incident CVD (heart disease or stroke) was ascertained from 2015 to 2020. Cox proportional hazards models estimated associations between trajectory groups and incident CVD, and Kaplan–Meier curves compared cumulative incidence across groups. **Results**: Stable MHNO accounted for 35.96% of the cohort, whereas the stable non-MHNO group accounted for 28.06%. After multivariable adjustment, the small obesity recovery group (*n* = 89) showed an elevated CVD risk estimate compared with the stable MHNO (HR 2.32, 95% CI 1.33–4.05), while stable non-MHNO group were associated with higher CVD risk (HR 1.72, 95% CI 1.36–2.18). Incident obesity showed elevated but non-significant risk (HR 1.71, 95% CI 0.94–3.10), and metabolic decline showed borderline significance (HR 1.36, 95% CI 1.00–1.85). Sex-stratified analyses showed heterogeneous risk patterns. **Conclusions**: Metabolic-obesity phenotypes are dynamic, but unfavorable phenotypes often persist. The obesity recovery group, defined using BMI, may represent a heterogeneous group and does not necessarily indicate true cardiometabolic recovery; the observed association should not be interpreted as evidence that weight loss itself increases CVD risk. These findings highlight the importance of jointly considering longitudinal changes in metabolic health and obesity when examining their associations with incident CVD.

## 1. Introduction

Cardiovascular disease (CVD), including heart disease and stroke, remains the leading cause of morbidity and mortality in China, particularly among middle-aged and older adults [1,2]. Obesity is a well-established and modifiable risk factor for CVD, but its cardiovascular consequences vary substantially according to metabolic health status. Accordingly, metabolic-obesity phenotypes, which jointly classify individuals by obesity and metabolic abnormality, have been widely used to characterize heterogeneous cardiometabolic risk beyond body mass index alone [3].

Accumulating evidence suggests that metabolically unhealthy obesity is associated with a higher risk of CVD, whereas the cardiovascular implications of metabolically healthy obesity remain controversial [4,5]. However, many previous studies have assessed metabolic-obesity phenotypes at a single time point, although recent research has begun to examine their longitudinal transitions. In particular, a previous CHARLS-based study by He et al. investigated transitions among metabolic–body weight phenotypes and their associations with incident CVD, showing that deterioration in obesity or metabolic health was associated with increased cardiovascular risk, whereas phenotype improvement was generally associated with lower risk [5]. Nevertheless, that study combined overweight and obesity into a single category. This approach may obscure the cardiovascular implications of specifically crossing the obesity threshold, particularly in Chinese adults for whom BMI ≥ 28 kg/m^2^ is used to define obesity. In addition, transition out of BMI-defined obesity may occur without concurrent recovery of metabolic health and therefore may not represent normalization of cardiovascular risk. The subsequent CVD risk associated with such apparent obesity recovery has not been clearly characterized.

Accordingly, using data from the China Health and Retirement Longitudinal Study (CHARLS), the present study aimed to evaluate dynamic changes in metabolic-obesity phenotypes among middle-aged and older adults and to further investigate the prospective associations between different phenotype trajectories and incident CVD. This study aimed to provide longitudinal evidence on the associations between metabolic-obesity phenotype trajectories and incident CVD in aging populations.

## 2. Materials and Methods

### 2.1. Study Design and Population

This study was based on data from the China Health and Retirement Longitudinal Study (CHARLS), a nationally representative prospective cohort study of Chinese adults aged 45 years and older and their household members. The cohort has been followed up over multiple waves, and its detailed design has been described previously [6]. Because detailed metabolic indicators were available at wave 1 (2011) and wave 3 (2015), wave 1 was designated as the phenotype baseline and wave 3 as the follow-up baseline/index date. Metabolic-obesity phenotype transitions were characterized between these two waves, and follow-up for incident CVD began at wave 3 and continued through wave 5 (2020) [7]. The CHARLS study was approved by the Ethics Committee of Peking University, and all participants provided written informed consent.

**Figure 1:**
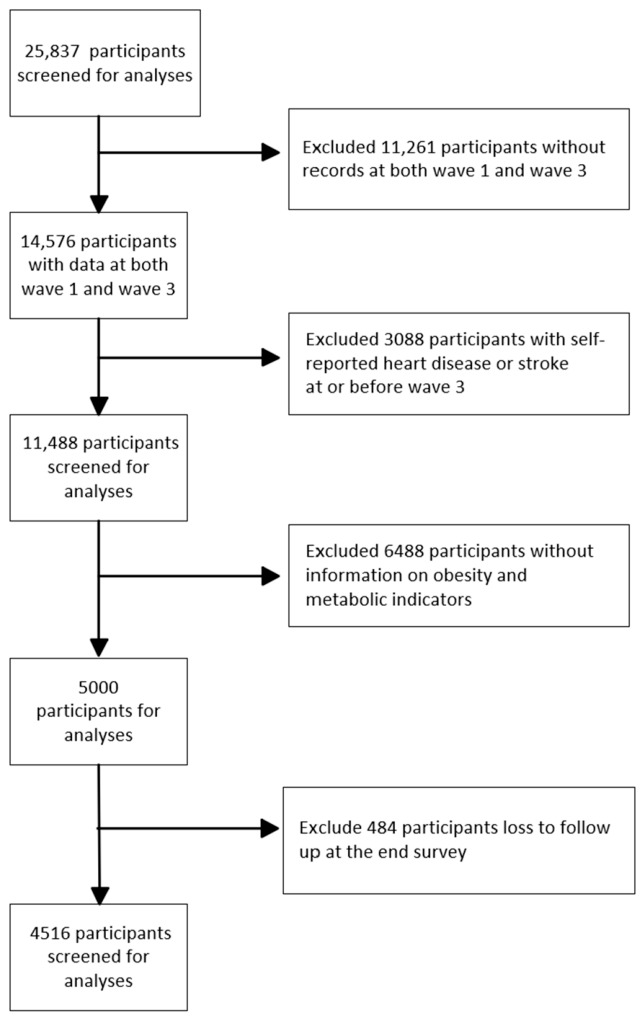
Flow chart of participant selection. Notes: Exclusions were applied sequentially in the order shown, and each participant was counted only once at the first exclusion criterion met.

Participants were eligible for inclusion if they met the following criteria: (1) they had records at both wave 1 and wave 3, which were required to construct metabolic-obesity phenotype transitions between the phenotype baseline and the follow-up baseline; 11,261 participants without records at both wave 1 and wave 3 were excluded; (2) they were free of CVD at the follow-up baseline/index date, defined as no self-reported physician-diagnosed heart disease or stroke at or before wave 3; 3088 participants with self-reported heart disease or stroke at or before wave 3 were excluded; (3) they had complete information for metabolic-obesity phenotype classification at both wave 1 and wave 3; 6488 participants were excluded; and (4) they had ascertainable CVD outcome data after wave 3; 484 participants were excluded. After these sequential exclusions, 4516 participants were ultimately included in the analysis, as shown in Figure 1.

### 2.2. Definition of Metabolic-Obesity Phenotypes

Obesity was defined as a body mass index (BMI) ≥ 28 kg/m^2^ according to the current Chinese criteria for adults [8,9]. BMI-defined obesity was used as the primary obesity criterion to maintain consistency with the conventional metabolic-obesity phenotype framework used in previous studies and to facilitate comparability with prior research in Chinese populations. Waist circumference, which better reflects central adiposity, was used as an alternative obesity definition in sensitivity analyses. Metabolic abnormality was defined as the presence of at least two of the following criteria: (1) triglycerides (TG) ≥ 1.7 mmol/L or use of lipid-lowering drugs; (2) high-density lipoprotein cholesterol (HDL-C) < 1.04 mmol/L for men, <1.29 mmol/L for women; (3) fasting blood glucose (FBG) ≥ 5.6 mmol/L or use of medications for diabetes; (4) systolic blood pressure ≥ 130 mmHg or diastolic blood pressure ≥ 85 mmHg or use of antihypertensive drugs.

By combining obesity status and metabolic status, participants were classified into four metabolic-obesity phenotypes: metabolically healthy non-obese (MHNO), metabolically unhealthy non-obese (MUNO), metabolically healthy obese (MHO), and metabolically unhealthy obese (MUO) [10]. To construct six mutually exclusive trajectory groups, we applied a hierarchical classification strategy. Changes in metabolic health status were prioritized: transitions from metabolically healthy to metabolically unhealthy phenotypes were classified as metabolic decline, whereas transitions from metabolically unhealthy to metabolically healthy phenotypes were classified as metabolic recovery, irrespective of concurrent changes in obesity status. Among participants whose metabolic health status remained unchanged, transitions from non-obese to obese status were classified as incident obesity, and transitions from obese to non-obese status were classified as obesity recovery. Participants who remained MHNO constituted the reference group. The constituent transitions of each trajectory group were defined according to the hierarchical classification strategy described above. This grouping strategy was intended to summarize the predominant direction of phenotype change and improve statistical precision, rather than to imply that all constituent transitions shared identical biological or clinical mechanisms.

### 2.3. Follow-Up and Definition of Outcomes

Follow-up for incident CVD began at the follow-up baseline/index date, wave 3 (2015), and ended at the first occurrence of CVD, the date of the last completed follow-up, or wave 5 (2020), whichever came first. In this study, incident CVD was defined as the first self-reported physician diagnosis of heart disease and/or stroke occurring after wave 3. Participants were considered to have experienced an outcome event when they first reported either heart disease or stroke during follow-up. Sensitivity analyses using separate outcome definitions are presented in Supplementary Figures S4 and S5. Participants who did not develop the outcome were censored at wave 5. The five waves of the CHARLS cohort were conducted in 2011, 2013, 2015, 2018, and 2020.

### 2.4. Covariates

Covariates were selected a priori based on their established associations with metabolic-obesity phenotypes and CVD and their availability in CHARLS. The primary model included age, sex, marital status, educational level, current smoking, and current drinking. Urban/rural residence was additionally included in a sensitivity analysis.

### 2.5. Statistical Analysis

All statistical analyses were performed using R 4.5.1. Continuous variables were expressed as means with standard deviations (SDs), and group comparisons were conducted using one-way analysis of variance. In addition, standardized mean differences (SMDs) were calculated to evaluate the magnitude of between-group differences. For comparisons involving more than two phenotype groups, the maximum pairwise SMD was reported. Categorical variables were presented as counts and percentages, with differences between groups assessed using the 2 test. The proportional hazards assumption was evaluated using Schoenfeld residuals, and no evident violation was found. Cox proportional hazards regression models were used to estimate hazard ratios (HRs) and 95% confidence intervals (CIs) for the associations between metabolic-obesity trajectory groups and incident CVD. Kaplan–Meier methods were used to plot cumulative incidence curves of CVD across trajectory groups, and the log-rank test was applied to compare differences between groups. Subgroup analyses were further performed according to sex. A two-tailed *p* value < 0.05 was considered statistically significant. Participants with missing data on key exposure variables, covariates, or outcome variables were excluded, and complete-case analysis was performed. Multiple imputation was not used.

Sensitivity analyses were performed to assess the robustness of the main findings. First, obesity was redefined using waist circumference instead of BMI [11], and the six metabolic-obesity trajectory groups were reconstructed using the same classification strategy. Second, all 16 original phenotype transitions between wave 1 and wave 3 were analyzed separately, with the stable MHNO-to-MHNO transition as the reference group. Because some individual transition groups had small sample sizes and wide confidence intervals, the 16-transition analyses were considered exploratory and are presented in Supplementary Figures S1–S3.

## 3. Results

### 3.1. Characteristics of the Study Population at Wave 3 (Follow-Up Baseline)

Table 1 summarizes the baseline characteristics at wave 3 according to metabolic-obesity phenotypes. Among 4516 participants, MUNO was the predominant phenotype, whereas MHO accounted for the smallest proportion. Baseline characteristics differed across phenotype groups. Compared with participants with the MHNO phenotype, those with MUO were relatively younger and more likely to be female. Significant between-group differences were observed for sex, marital status, current smoking, current drinking, BMI, systolic blood pressure, diastolic blood pressure, triglycerides, HDL-C, and fasting glucose, whereas educational level did not differ significantly across groups. Consistent with the phenotype definitions, participants with metabolically unhealthy phenotypes, particularly those with concomitant obesity, exhibited less favorable cardiometabolic profiles. In summary, MUNO was the most common phenotype, whereas MHO was the least common phenotype. Overall, the prevalence of BMI-defined obesity in the study population was 11.2%, calculated by combining participants with MHO and MUO phenotypes.

### 3.2. Longitudinal Transitions in Metabolic-Obesity Phenotypes

**Figure 2:**
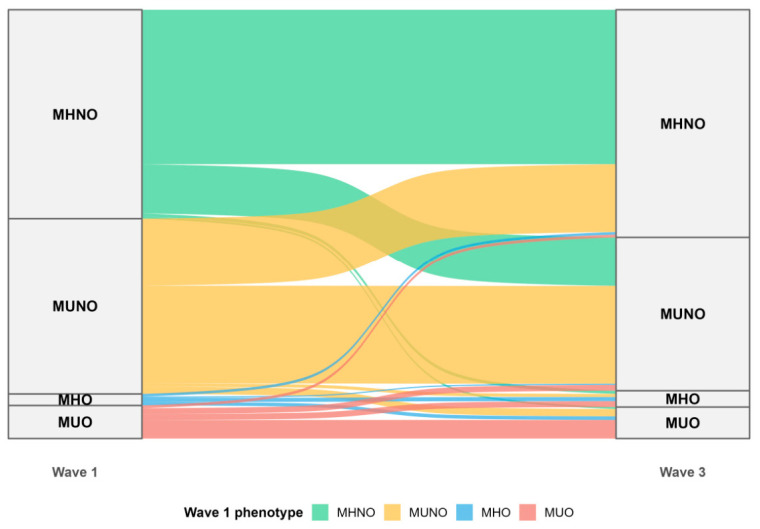
Sankey plot of longitudinal transitions in metabolic-obesity phenotypes in the CHARLS Cohort, 2011–2015.

Table 2 summarizes longitudinal changes in metabolic-obesity phenotypes from 2011 to 2015. Stable MHNO was the largest single trajectory group, accounting for 35.96% of the cohort, whereas the stable non-MHNO group accounted for 28.06%. Within the stable non-MHNO group, the MUNO-to-MUNO transition was the dominant pathway, accounting for 22.74% of the cohort. Metabolic recovery occurred in 18.51% of participants, mainly through the transition from MUNO to MHNO, whereas metabolic decline occurred in 13.04% of participants, largely driven by the transition from MHNO to MUNO. In contrast, transitions involving obesity status alone were relatively uncommon, including incident obesity and obesity recovery, which accounted for 2.46% and 1.97% of participants, respectively. Metabolic recovery (18.51%) was more common than obesity recovery (1.97%). Figure 2 visually illustrates the transition pathways of metabolic-obesity phenotypes. The stable MHNO group accounted for the largest proportion, whereas MHO appeared to be unstable, with only a small number of participants remaining in the same phenotype. Overall, these findings suggest that changes in metabolic health were more frequent than changes in obesity status alone, although persistent unfavorable phenotype patterns remained common.

### 3.3. Association of Metabolic-Obesity Trajectories with Incident Cardiovascular Disease

As shown in Table 3, CVD incidence rates differed across metabolic-obesity trajectory groups. The obesity recovery group had the highest incidence rate, at 64.1 per 1000 person-years, followed by incident obesity and stable non-MHNO groups, with incidence rates of 53.3 and 50.9 per 1000 person-years, respectively. In contrast, the stable MHNO group had the lowest incidence rate, at 30.6 events per 1000 person-years.

**Figure 3:**
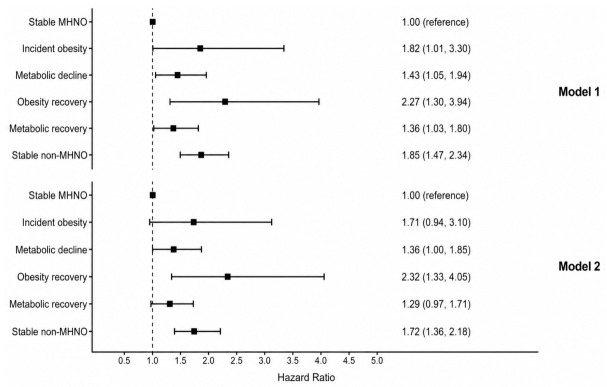
Hazard ratios for incident cardiovascular disease according to metabolic-obesity trajectory groups. Notes: Hazard ratios (*HRs*) and 95% confidence intervals (*CIs*) for incident cardiovascular disease (CVD) across six metabolic-obesity trajectory groups, estimated using Cox proportional hazards models. Stable MHNO served as the reference group. Model 1 was unadjusted, whereas Model 2 was adjusted for age, sex, marital status, educational level, smoking, and drinking. Additional adjustment for urban/rural residence yielded generally consistent directions of association, although the magnitude and statistical significance of some estimates changed (Supplementary Figure S6).

**Figure 4:**
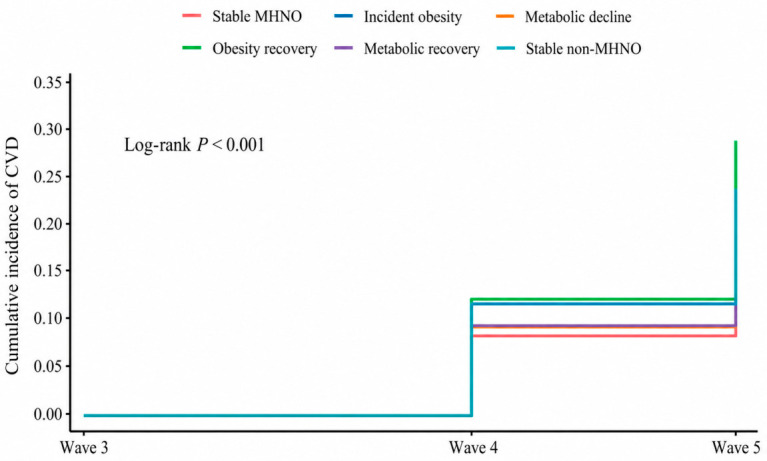
Kaplan–Meier curves for incident cardiovascular disease according to metabolic-obesity trajectory groups. Notes: The curves show the cumulative incidence of cardiovascular disease (CVD) across the six trajectory groups. Differences between groups were assessed using the log-rank test. Because exact dates of CVD onset were unavailable, follow-up time was assigned according to the first survey wave at which heart disease or stroke was reported. Wave 3 (2015) was treated as time zero; participants who first reported CVD at wave 4 (2018) or wave 5 (2020) were assigned follow-up times of 3 and 5 years, respectively. Participants without incident CVD were censored at wave 5.

Consistent with these findings, the Cox regression results are shown in Figure 3, compared with the Stable MHNO group, obesity recovery and stable non-MHNO groups were significantly associated with a higher risk of incident CVD after multivariable adjustment, with HRs of 2.32 (95% CI, 1.33–4.05) and 1.72 (95% CI, 1.36–2.18), respectively. Incident obesity showed an elevated but statistically non-significant risk estimate (HR, 1.71; 95% CI, 0.94–3.10). Metabolic decline showed a borderline positive association (HR, 1.36; 95% CI, 1.00–1.85), whereas metabolic recovery was not statistically significant (HR, 1.29; 95% CI, 0.97–1.71). As shown in Figure 3 and Figure 4, the adjusted risk estimates and cumulative incidence curves consistently indicated higher CVD risk in the obesity recovery and stable non-MHNO groups, whereas the evidence for incident obesity and metabolic-status transitions alone was less robust. Kaplan–Meier analysis demonstrated that cumulative CVD incidence differed significantly among the six trajectory groups (log-rank *p* < 0.001). Additional clinical characteristics of participants in the obesity recovery group are shown in Supplementary Table S1. This descriptive analysis was conducted to provide clinical context for interpreting the elevated CVD risk observed in this small subgroup.

### 3.4. Subgroup and Sensitivity Analyses

**Figure 5:**
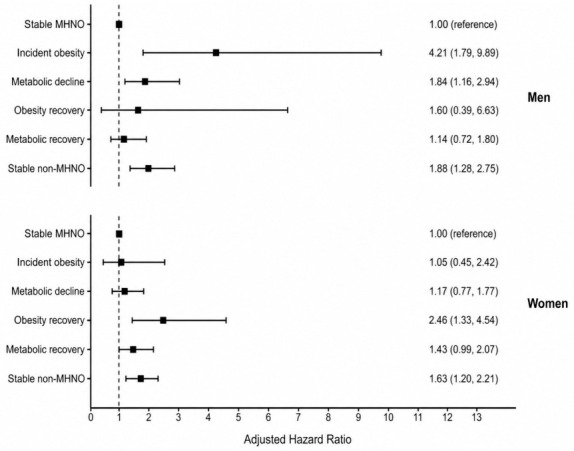
Subgroup analysis by sex of the associations between metabolic-obesity trajectory groups and incident cardiovascular disease. Notes: Forest plots showing adjusted hazard ratios (*HRs*) and 95% confidence intervals (*CIs*) for incident cardiovascular disease (CVD) across metabolic-obesity trajectory groups in women and men separately. Stable MHNO served as the reference group. Cox proportional hazards models were adjusted for age, marital status, educational attainment, smoking, and drinking. The interaction between sex and metabolic-obesity trajectory group was not statistically significant (*P* for interaction = 0.299).

Exploratory sex-stratified analyses showed some apparent differences in the associations between metabolic-obesity groups and incident CVD (Figure 5). Among men, incident obesity, metabolic decline, and stable non-MHNO groups were significantly associated with increased CVD risk compared with stable MHNO, with adjusted HRs of 4.21 (95% CI, 1.79–9.89), 1.84 (95% CI, 1.16–2.94), and 1.88 (95% CI, 1.28–2.75), respectively. Obesity recovery and metabolic recovery were not statistically significant in men. Among women, the obesity recovery and stable non-MHNO groups were significantly associated with higher CVD risk, with adjusted HRs of 2.46 (95% CI, 1.33–4.54) and 1.63 (95% CI, 1.20–2.21), respectively, while metabolic recovery showed a borderline association. However, the formal interaction test did not show a statistically significant interaction between sex and metabolic-obesity trajectory group (*P* for interaction = 0.299), indicating limited statistical evidence that these associations differed by sex. The numbers of participants and CVD events in each metabolic-obesity trajectory group stratified by sex are presented in Supplementary Table S2.

Sensitivity analyses showed generally consistent directions of association. When obesity was redefined using waist circumference, the overall association pattern remained broadly consistent with the BMI-based analysis. Analyses of the original 16 phenotype transitions and separate outcomes of heart disease and stroke also showed generally similar patterns. After additional adjustment for urban/rural residence, the overall pattern of associations remained broadly consistent, although the magnitude and statistical significance of some estimates changed. Detailed results are presented in Supplementary Figures S1–S6.

## 4. Discussion

In this prospective cohort study of middle-aged and older Chinese adults, we found that metabolic-obesity phenotypes were dynamic over time, while unfavorable phenotype patterns showed substantial persistence. Stable MHNO was the largest trajectory group, whereas the stable non-MHNO group also accounted for a considerable proportion of the cohort. Compared with stable MHNO, the stable non-MHNO group was associated with a higher risk of subsequent CVD, while the small obesity recovery group showed an elevated risk estimate that warrants cautious interpretation. Incident obesity showed an elevated but statistically non-significant association, and metabolic decline showed a borderline association. These findings indicate that the cardiovascular implications of phenotype change depend on which component improves. In particular, transition below the BMI-defined obesity threshold should not be considered equivalent to cardiometabolic recovery when metabolic abnormalities persist [12].

The relatively low prevalence of obesity in this cohort should also be noted. Using the Chinese BMI criterion of ≥28 kg/m^2^, 11.2% of participants were classified as obese, which is consistent with epidemiological evidence from older Chinese adults but lower than that reported in many Western populations [13]. This population-specific feature may reflect differences in anthropometric characteristics, BMI distribution, body composition, and obesity classification criteria. As a result, non-obese but metabolically unhealthy phenotypes were relatively common, whereas obesity-related phenotypes and obesity-status transitions were less frequent. Therefore, our findings are most applicable to middle-aged and older Chinese adults and should be generalized cautiously to younger populations or populations with a substantially higher prevalence of obesity. BMI may also underestimate adiposity in middle-aged and older Chinese adults, because abdominal obesity and visceral fat accumulation can occur even among individuals with normal BMI [14,15]. Consequently, some participants with central obesity may have been classified as BMI-defined non-obese, which may partly contribute to the high proportion of MUNO and the low frequency of obesity-related transitions. This issue is particularly relevant to the obesity recovery group, which was defined using BMI, because transition out of BMI-defined obesity may not necessarily reflect true reductions in central adiposity or cardiometabolic risk. Nevertheless, the waist circumference-based sensitivity analysis showed broadly consistent association patterns, partially supporting the robustness of the main findings.

The increased CVD risk observed in the stable non-MHNO group may reflect prolonged exposure to adverse cardiometabolic conditions. In our study, the stable non-MHNO group included stable MUNO, MUO, and MHO participants. Therefore, its excess risk is more likely to reflect the long-term maintenance of a non-ideal cardiometabolic state, driven either by sustained metabolic disturbance, persistent obesity burden, or both. For individuals with stable MUNO or MUO, prolonged exposure to hypertension, dysglycemia, dyslipidemia, and related metabolic stress may promote endothelial dysfunction, chronic low-grade inflammation, insulin resistance, and atherosclerotic progression, thereby increasing the likelihood of cardiovascular events [16,17]. Meanwhile, the inclusion of stable MHO suggests that even in the absence of overt metabolic abnormality, persistent obesity itself may still confer excess cardiovascular risk through mechanisms not fully captured by conventional metabolic markers, such as excess adipose tissue burden, adverse fat distribution, hemodynamic overload, and subclinical inflammation [18,19].

The small obesity recovery group showed an elevated CVD risk estimate; however, this finding requires cautious interpretation because the group comprised only 89 participants and 27 CVD events and was likely heterogeneous. This result should not be interpreted as evidence that weight reduction is harmful [20]. Rather, it suggests that BMI-defined transition out of obesity may not necessarily indicate cardiometabolic recovery. In this group, most participants transitioned from MUO to MUNO, meaning that they were no longer classified as obese according to BMI but remained metabolically unhealthy. Therefore, the elevated CVD risk in this group may reflect residual metabolic dysfunction, prior exposure to obesity-related cardiometabolic injury, persistent visceral adiposity or ectopic fat deposition not captured by BMI, or heterogeneous weight-loss processes in middle-aged and older adults [21,22,23,24]. Although the obesity recovery group showed higher BMI, waist circumference, blood pressure, and diabetes prevalence at wave 3, available indicators of chronic disease and functional status did not differ significantly from other trajectory groups. Nevertheless, because information on intentionality of weight loss, frailty, sarcopenia, malignancy severity, chronic kidney disease severity, and other catabolic conditions was limited, illness-related weight loss and reverse causation cannot be excluded. Therefore, the elevated risk in the obesity recovery group should be interpreted as residual or heterogeneous risk after BMI-defined transition out of obesity, rather than as evidence against weight reduction. These findings highlight the need to distinguish between obesity recovery based on BMI-defined obesity status and true cardiometabolic recovery based on improvement in metabolic risk factors.

Sex-stratified analyses suggested some trajectory-specific differences in the associations between metabolic-obesity trajectories and incident CVD. Such differences may be biologically plausible given previously reported sex differences in visceral fat accumulation, metabolic responses to adiposity, lifestyle-related risk factors, and age-related or postmenopausal changes in body composition [25,26,27,28]. However, the formal interaction test between sex and metabolic-obesity trajectory was not statistically significant. Therefore, these sex-specific findings should be interpreted as exploratory and hypothesis-generating rather than definitive evidence of sex-related effect modification.

Our findings are broadly consistent with previous studies showing that metabolically unhealthy obesity and transitions from healthy to unhealthy metabolic status are associated with increased cardiovascular risk [29,30]. Our study has important clinical and public health implications. First, cardiovascular risk assessment in middle-aged and older adults should move beyond BMI alone and incorporate repeated assessment of metabolic health indicators. Second, individuals who transition out of obesity according to BMI should not automatically be considered low risk, especially if hypertension, dysglycemia, dyslipidemia, or other metabolic abnormalities remain present. Third, the consistency of waist circumference-based sensitivity analyses further supports the importance of considering central obesity and body fat distribution in addition to BMI. Longitudinal monitoring of both obesity status and metabolic health may help identify high-risk individuals who require more intensive prevention and management.

This study has several strengths. First, we applied the Chinese obesity-specific cutoff of BMI ≥ 28 kg/m^2^ rather than combining overweight and obesity, thereby allowing transitions into and out of obesity to be evaluated separately. Second, the six-trajectory framework distinguished changes in obesity status from changes in metabolic health, particularly obesity recovery from metabolic recovery, while analyses of the 16 original transitions retained information on the specific transition pathways. Third, waist circumference-based classification and separate analyses of heart disease and stroke were performed to evaluate the robustness of the main findings. The nationally representative CHARLS cohort further supports the relevance of these observations to middle-aged and older Chinese adults. Nevertheless, several limitations should also be acknowledged. First, incident CVD was ascertained on the basis of self-reported physician diagnosis, which may have led to outcome misclassification. Second, phenotype trajectories were defined using only two time points, which may not have fully captured more complex long-term transition patterns. Third, obesity was primarily defined using BMI, which does not directly measure adiposity or body composition and cannot distinguish fat mass from lean mass, visceral fat, sarcopenic obesity, ectopic fat deposition, or age-related body composition changes. Although waist circumference was used as an alternative definition in sensitivity analyses, it remains an indirect anthropometric measure. Because body composition data, such as bioelectrical impedance analysis-derived fat mass and lean mass, were unavailable in the CHARLS waves used for this study, some participants with sarcopenic obesity or excess visceral adiposity despite normal BMI may have been misclassified as non-obese. This may have influenced phenotype classification and the observed associations with CVD risk, although the waist circumference-based sensitivity analysis showed broadly consistent results. Finally, although major demographic and lifestyle factors were adjusted for and urban/rural residence was additionally considered in a sensitivity analysis, residual confounding from unmeasured or incompletely measured factors, such as diet, physical activity, socioeconomic status, and medication use, cannot be excluded.

## 5. Conclusions

In conclusion, metabolic-obesity phenotypes in the CHARLS cohort were dynamic over time, while unfavorable phenotype patterns showed substantial persistence. The stable non-MHNO group was associated with a higher risk of subsequent CVD. The small obesity recovery group, defined using BMI, also showed an elevated risk estimate; however, this category was heterogeneous and may have included intentional or unintentional weight loss and changes in body composition. This finding should not be interpreted as evidence that obesity recovery itself increases CVD risk. Rather, transition below the BMI obesity threshold does not necessarily indicate true cardiometabolic recovery, particularly when metabolic abnormalities persist. Exploratory sex-stratified analyses did not provide statistically significant evidence of sex-related effect modification.

## Figures and Tables

**Table 1:** Characteristics of baseline data at wave 3 stratified by metabolic-obesity phenotype.

Variables	All Participants	MHNO	MUNO	MHO	MUO	*c2/F*	*p* Value	Max SMD
	(*n* = 4516)	(*n* = 2393)	(*n* = 1619)	(*n* = 173)	(*n* = 331)			
Age, mean (SD), years	61.50 ± 8.71	61.52 ± 8.58	62.35 ± 8.99	56.87 ± 6.98	59.60 ± 7.94	27.122	<0.001	0.68
Sex, *n* (%)						73.082	<0.001	0.457
Female	2513 (55.65)	1209 (50.52)	958 (59.17)	107 (61.85)	239 (72.21)			
Male	2003 (44.35)	1184 (49.48)	661 (40.83)	66 (38.15)	92 (27.79)			
Education, *n* (%)						12.323	0.196	0.213
Below primary school	2285 (50.6)	1196 (50.0)	836 (51.6)	75 (43.4)	178 (53.8)			
Primary school	994 (22.0)	539 (22.5)	352 (21.7)	41 (23.7)	62 (18.7)			
Middle school	865 (19.2)	453 (18.9)	305 (18.8)	36 (20.8)	71 (21.5)			
High school or above	372 (8.2)	205 (8.6)	126 (7.8)	21 (12.1)	20 (6.0)			
Marital status, *n* (%)						15.026	0.002	0.194
Married or partnered	3773 (83.5)	2025 (84.6)	1310 (80.9)	147 (85.0)	291 (87.9)			
Other marital status	743 (16.5)	368 (15.4)	309 (19.1)	26 (15.0)	40 (12.1)			
Current smoking, *n* (%)						58.131	<0.001	0.428
No	3304 (73.21)	1658 (69.37)	1218 (75.23)	141 (81.50)	287 (86.71)			
Yes	1212 (26.79)	732 (30.63)	401 (24.77)	32 (18.50)	44 (13.29)			
Current drinking, *n* (%)						24.169	<0.001	0.271
No	2954 (65.47)	1502 (62.79)	1085 (67.10)	118 (68.60)	249 (75.23)			
Yes	1562 (34.53)	890 (37.21)	532 (32.90)	54 (31.40)	82 (24.77)			
BMI, mean (SD), kg/m^2^	23.70 ± 3.49	22.30 ± 2.64	23.77 ± 2.53	30.06 ± 1.83	30.35 ± 2.15	97.659	<0.001	0.572
SBP, mean (SD), mmHg	126.73 ± 19.57	120.53 ± 17.19	134.22 ± 19.94	125.47 ± 16.59	135.65 ± 19.32	207.268	<0.001	0.827
DBP, mean (SD), mmHg	74.72 ± 11.61	71.53 ± 10.40	77.94 ± 11.92	76.28 ± 10.18	81.24 ± 11.89	151.287	<0.001	0.869
Triglycerides, mean (SD), mg/dL	142.10 ± 90.75	98.55 ± 39.81	194.21 ± 107.51	119.26 ± 53.45	213.99 ± 102.45	610.613	<0.001	1.485
HDL-C, mean (SD), mg/dL	51.91 ± 11.83	55.87 ± 11.56	47.33 ± 10.87	52.05 ± 9.29	45.54 ± 8.04	233.732	<0.001	1.038
Fasting glucose, mean (SD), mg/dL	102.90 ± 33.24	93.86 ± 21.49	115.35 ± 43.18	92.53 ± 14.13	112.82 ± 31.83	166.584	<0.001	0.824

Notes: Continuous variables are presented as mean ± SD and were compared using one-way ANOVA. Categorical variables are presented as *n* (%) and were compared using chi-square tests. Max SMD represents the maximum pairwise standardized mean difference across the four phenotype groups. An SMD ≥ 0.10 was considered to indicate a meaningful between-group difference. Lipid and glucose values are presented in mg/dL for descriptive purposes; phenotype classification was based on mmol/L cutoffs after unit conversion. BMI, body mass index; SBP, systolic blood pressure; DBP, diastolic blood pressure; HDL-C, high-density lipoprotein cholesterol; SD, standard deviation; SMD, standardized mean difference.

**Table 2:** Distribution of the 16 metabolic-obesity phenotype transitions across six trajectory groups.

Group	Wave 1 (2011)		Wave 3 (2015)	Transfer Path	Total, *n*(%)
Stable MHNO	MHNO		MHNO	MHNO → MHNO	1624 (35.96%)
Total	---	---	---	---	1624 (35.96%)
Incident obesity	MHNO		MHO	MHNO → MHO	34 (0.75%)
Incident obesity	MUNO		MUO	MUNO → MUO	77 (1.71%)
Total	---	---	---	---	111 (2.46%)
Metabolic decline	MHNO		MUNO	MHNO → MUNO	518 (11.47%)
Metabolic decline	MHNO		MUO	MHNO → MUO	21 (0.47%)
Metabolic decline	MHO		MUNO	MHO → MUNO	13 (0.29%)
Metabolic decline	MHO		MUO	MHO → MUO	37 (0.82%)
Total	---	---	---	---	589 (13.04%)
Obesity recovery	MHO		MHNO	MHO → MHNO	28 (0.62%)
Obesity recovery	MUO		MUNO	MUO → MUNO	61 (1.35%)
Total	---	---	---	---	89 (1.97%)
Metabolic recovery	MUNO		MHNO	MUNO → MHNO	712 (15.77%)
Metabolic recovery	MUNO		MHO	MUNO → MHO	33 (0.73%)
Metabolic recovery	MUO		MHNO	MUO → MHNO	29 (0.64%)
Metabolic recovery	MUO		MHO	MUO → MHO	62 (1.37%)
Total	---	---	---	---	836 (18.51%)
Stable non-MHNO	MHO		MHO	MHO → MHO	44 (0.97%)
Stable non-MHNO	MUNO		MUNO	MUNO → MUNO	1027 (22.74%)
Stable non-MHNO	MUO		MUO	MUO → MUO	196 (4.34%)
Total	---	---	---	---	1267 (28.06%)

**Table 3:** Incidence rates and hazard ratios for incident cardiovascular disease according to metabolic-obesity trajectory groups.

Trajectory Group	*N*	CVD Events (*n*)	Person-Years	Incidence Rate (per 1000 Person-Years)	Model 1 *HR* (95% *CI*)	Model 2 *HR* (95% *CI*)
Stable MHNO	1624	240	7840	30.6	1.00 (reference)	1.00 (reference)
Incident obesity	111	28	525	53.3	1.82 (1.01–3.30)	1.71 (0.94–3.10)
Metabolic decline	589	106	2831	37.4	1.43 (1.05–1.94)	1.36 (1.00–1.85)
Obesity recovery	89	27	421	64.1	2.27 (1.30–3.94)	2.32 (1.33–4.05)
Metabolic recovery	836	178	4012	44.4	1.36 (1.03–1.80)	1.29 (0.97–1.71)
Stable non-MHNO	1267	307	6033	50.9	1.85 (1.47–2.34)	1.72 (1.36–2.18)

Notes: Person-years were calculated from wave 3 (2015) to the first occurrence of cardiovascular disease (CVD), the last completed follow-up, or wave 5 (2020), whichever came first. Incidence rates were expressed per 1000 person-years. Model 1 was unadjusted, whereas Model 2 was adjusted for age, sex, marital status, educational level, smoking, drinking. Stable MHNO served as the reference group.

## Data Availability

The data used in this study were obtained from the China Health and Retirement Longitudinal Study (CHARLS) and are available at https://charls.charlsdata.com/ upon registration and approval by the CHARLS research team.

## Supplementary Materials

The following supporting information can be downloaded at: https://www.mdpi.com/article/10.3390/metabo16070510/s1, Table S1: Clinical characteristics of participants in the obesity recovery group compared with other trajectory groups; Table S2: Number of participants and CVD events by sex and metabolic-obesity trajectory group; Figure S1: Forest plot of BMI-defined 16 metabolic-obesity phenotype transitions and incident cardiovascular disease; Figure S2: Forest plot of waist circumference-defined metabolic-obesity trajectory groups and incident cardiovascular disease; Figure S3: Forest plot of waist circumference-defined 16 metabolic-obesity phenotype transitions and incident cardiovascular disease; Figure S4: Forest plot of metabolic-obesity trajectory groups and incident heart disease; Figure S5: Forest plot of metabolic-obesity trajectory groups and incident stroke. Figure S6: Sensitivity analysis of the associations between metabolic-obesity trajectory groups and incident cardiovascular disease with additional adjustment for urban/rural residence.

## Abbreviations

The following abbreviations are used in this manuscript:

ANOVA	Analysis of variance
BMI	Body mass index
CHARLS	China Health and Retirement Longitudinal Study
CI	Confidence interval
CVD	Cardiovascular disease
DBP	Diastolic blood pressure
FBG	Fasting blood glucose
HDL-C	High-density lipoprotein cholesterol
HR	Hazard ratio
ID	Identification number
MHO	Metabolically healthy obese
MHNO	Metabolically healthy non-obese
MUO	Metabolically unhealthy obese
MUNO	Metabolically unhealthy non-obese
SBP	Systolic blood pressure
SD	Standard deviation
SMD	Standardized mean difference
TG	Triglycerides
