# Supplementary material for: Longitudinal Transitions of Metabolic-Obesity Phenotypes and Subsequent Cardiovascular Disease Risk: A Prospective Analysis of the CHARLS Cohort
Source: Metabolites. 2026 Jul 21;16(7):510. doi: 10.3390/metabo16070510 (PMC13414334; doi:10.3390/metabo16070510)
Supplement: Supplementary file 1 [file metabolites-16-00510-s001.zip › metabolites-4423993-supplementary.pdf]

Supplementary Materials

Supplementary Table S1. Clinical characteristics of participants in the obesity recovery group compared with other trajectory groups.

Variable	Obesity recovery (n = 89)	Other trajectory groups (n = 4427)	*P* value
Age, years	58.27 ± 8.05	61.56 ± 8.71	<0.001
BMI, kg/m²	26.16 ± 2.07	24.22 ± 1.48	<0.001
Waist circumference, cm	89.59 ± 14.43	84.30 ± 13.48	<0.001
Systolic blood pressure, mmHg	134.38 ± 20.30	126.58 ± 19.53	<0.001
Diastolic blood pressure, mmHg	78.60 ± 10.83	74.64 ± 11.61	<0.001
Diabetes, n (%)	22 (25.6)	342 (7.9)	<0.001
Cancer, n (%)	2 (2.2)	52 (1.2)	0.294
Kidney disease, n (%)	5 (5.8)	353 (8.1)	0.566
Chronic lung disease, n (%)	10 (11.4)	534 (12.2)	0.942
Stomach or digestive disease, n (%)	24 (27.3)	1358 (31.1)	0.513
Psychiatric disease, n (%)	1 (1.1)	80 (1.8)	1.000
Asthma, n (%)	4 (4.5)	211 (4.8)	1.000
ADL limitation, n (%)	17 (19.5)	830 (18.8)	0.973
IADL limitation, n (%)	15 (16.9)	905 (20.5)	0.483
Fall, n (%)	31 (34.8)	1515 (34.2)	0.997

Notes: Values are presented as mean ± SD for continuous variables and n (%) for categorical variables. Clinical characteristics were assessed at wave 3, the start of follow-up. The obesity recovery group inclu ded participants who transitioned from MHO to MHNO or from MUO to MUNO between wave 1 and wave 3. Other trajectory groups included all remaining metabolic-obesity trajectory groups. P values were calculated using Wilcoxon rank-sum tests for continuous variables and chi-square or Fisher's exact tests for categorical variables, as appropriate. BMI, body mass index; ADL, activities of daily living; IADL, in strumental activities of daily living; MHNO, metabolically healthy non-obese; MUNO, metabolically unhea lthy non-obese; MHO, metabolically healthy obese; MUO, metabolically unhealthy obese.Percentages were calculated using the number of participants with non-missing data for each variable as the denominator; therefore, denominators may vary across variables.

Supplementary Table S2. Number of participants and CVD events by sex and metabolic-obesity trajectory group.

Group	Male participants, n (%)	Male CVD events, n (%)	Female participants, n (%)	Female CVD events, n (%)

Stable MHNO	818 (50.4)	107 (13.1)	806 (49.6)	133 (16.5)
Incident obesity	33 (29.7)	7 (21.2)	78 (70.3)	21 (26.9)
Metabolic decline	248 (42.1)	48 (19.4)	341 (57.9)	58 (17.0)
Obesity recovery	25 (28.1)	8 (32.0)	64 (71.9)	19 (29.7)
Metabolic recovery	401 (48.0)	73 (18.2)	435 (52.0)	105 (24.1)
Stable non-MHNO	478 (37.7)	108 (22.6)	789 (62.3)	199 (25.2)

Values are presented as n (%). Percentages of male and female participants were calculated using the tot al number of participants in each trajectory group as the denominator. Percentages of CVD events were c alculated using the number of participants of the corresponding sex within each trajectory group as the d enominator. CVD events were defined as incident heart disease and/or stroke occurring after wave 3. Met abolic-obesity trajectory groups were defined according to phenotype transitions between wave 1 and wav e 3. CVD, cardiovascular disease; MHNO, metabolically healthy non-obese.

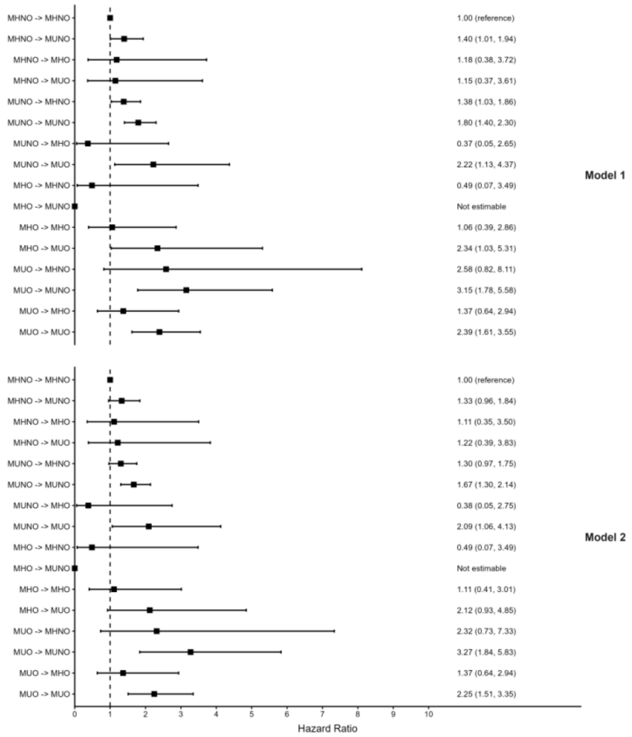

Supplementary Figure S1. Forest plot of BMI-defined 16 metabolic-obesity phenotype transitions and incident cardiovascular disease. Hazard ratios (*HRs*) and 95% confidence intervals (*CIs*) were estimated using Cox proportional hazards models. The MHNO-to-MHNO transition was used as the reference group. Model 1 was unadjusted, and Model 2 was adjusted for age, sex, marital status, educational level, current smoking, and current drinking. Because several transition groups had small sample sizes and wide CIs, these results should be interpreted as exploratory. BMI, body mass index; CVD, cardiovascular disease; MHNO, metabolically healthy non-obese; MUNO, metabolically unhealthy non-obese; MHO, metabolically healthy obese; MUO, metabolically

unhealthy obese.

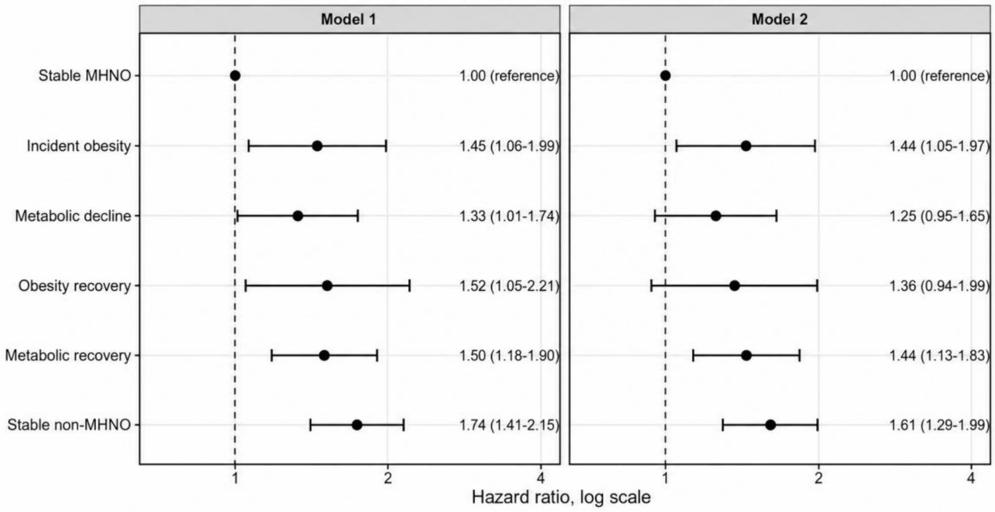

Supplementary Figure S2. Forest plot of waist circumference-defined metabolic-obesity trajectory groups and incident cardiovascular disease. Obesity was redefined using waist circumference, and the six metabolic-obesity trajectory groups were reconstructed using the same classification strategy as in the main BMI-based analysis. HRs and 95% CIs were estimated using Cox proportional hazards models, with stable MHNO as the reference group. Model 1 was unadjusted, and Model 2 was adjusted for age, sex, marital status, educational level, current smoking, and current drinking. CVD, cardiovascular disease; HR, hazard ratio; CI, confidence interval; MHNO, metabolically healthy non-obese.

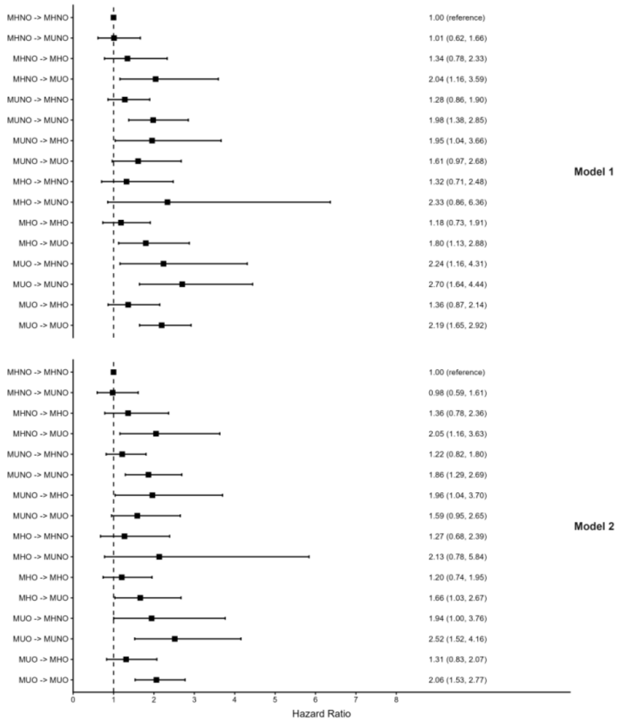

Supplementary Figure S3. Forest plot of waist circumference-defined 16 metabolic-obesity phenotype

transitions and incident cardiovascular disease. Sixteen phenotype transitions were reconstructed using waist circumference-defined obesity and metabolic health status. HRs and 95% CIs were estimated using Cox proportional hazards models, with the MHNO-to-MHNO transition as the reference group. Model 1 was unadjusted, and Model 2 was adjusted for age, sex, marital status, educational level, current smoking, and current drinking. Because several transition groups had small sample sizes and wide CIs, these results should be interpreted as exploratory. CVD, cardiovascular disease; HR, hazard ratio; CI, confidence interval; MHNO, metabolically healthy non-obese; MUNO, metabolically unhealthy non-obese; MHO, metabolically healthy obese; MUO, metabolically unhealthy obese.

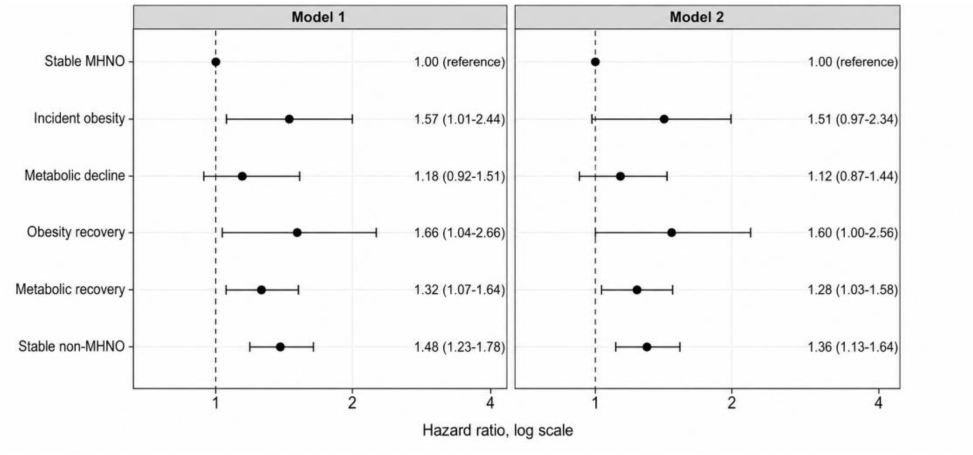

Supplementary Figure S4. Forest plot of metabolic-obesity trajectory groups and incident heart disease. HRs and 95% CIs for incident heart disease were estimated using Cox proportional hazards models according to metabolic-obesity trajectory groups. Stable MHNO was used as the reference group. Model 1 was unadjusted, and Model 2 was adjusted for age, sex, marital status, educational level, current smoking, and current drinking. This sensitivity analysis evaluated heart disease separately from the composite CVD outcome. HR, hazard ratio; CI, confidence interval; CVD, cardiovascular disease; MHNO, metabolically healthy non-obese.

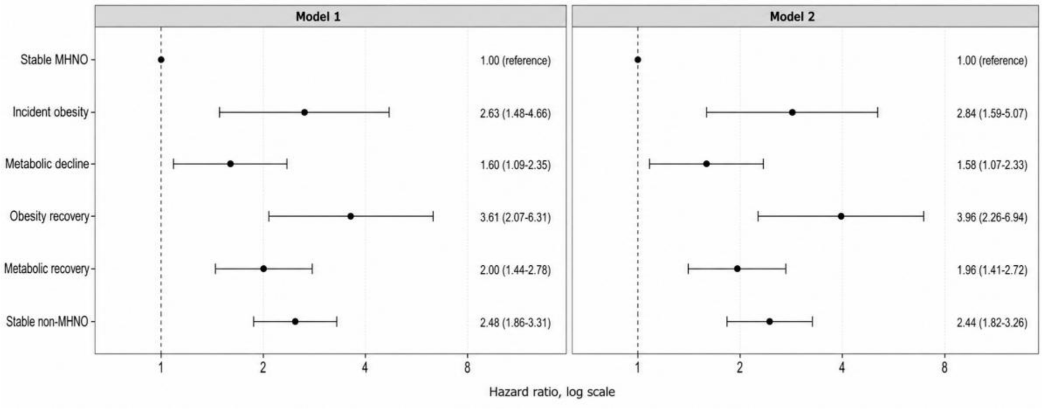

Supplementary Figure S5. Forest plot of metabolic-obesity trajectory groups and incident stroke. HRs and 95% CIs for incident stroke were estimated using Cox proportional hazards models according to metabolic-obesity trajectory groups. Stable MHNO was used as the reference group. Model 1 was unadjusted, and Model 2 was adjusted for age, sex, marital status, educational level, current smoking, and current drinking. This sensitivity analysis evaluated stroke separately from the composite CVD outcome. HR, hazard ratio; CI, confidence interval; CVD, cardiovascular disease; MHNO, metabolically healthy non-obese.

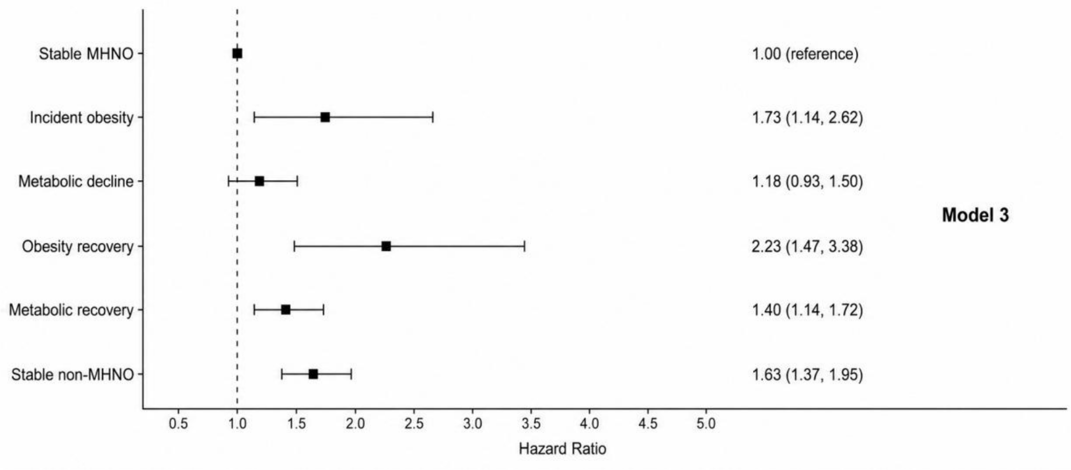

Supplementary Figure S6. Sensitivity analysis of the associations between metabolic-obesity trajectory groups and incident cardiovascular disease with additional adjustment for urban/rural residence.

Notes:Hazard ratios (HRs) and 95% confidence intervals (CIs) for incident cardiovascular disease were estimated using Cox proportional hazards models. Stable MHNO served as the reference group. Model 3 was adjusted for age, sex, marital status, educational level, current smoking, current drinking, and urban/rural residence. HR, hazard ratio; CI, confidence interval; CVD, cardiovascular disease; MHNO, metabolically healthy non-obese.
